# Clinical effects of 3D printing-assisted posterolateral incision in the treatment of ankle fractures involving the posterior malleolus

**DOI:** 10.3389/fsurg.2023.1176254

**Published:** 2023-05-24

**Authors:** Hongming Zheng, Yan Xia, Xiaohui Ni, Jieshi Wu, Yankun Li, Pengpeng Zhang, Xinglin Wu, Kaihang Lu, Quanming Zhao

**Affiliations:** ^1^ Department of Orthopedic Surgery, Affiliated Danyang Hospital of Nantong University, Danyang, China; ^2^ Department of Orthopedics, Dafeng People’s Hospital, Yancheng, China; ^3^ Department of Orthopaedics, Affiliated Hospital of Jiangnan University, Wuxi, China; ^4^ Department of Orthopaedics, Guizhou Provincial People's Hospital, Guiyang, China

**Keywords:** posterolateral approach, trimalleolar fracture, internal fixation, fractures - bone, 3D printing

## Abstract

**Objective:**

To explore the clinical outcomes of a 3D printing-assisted posterolateral approach for the treatment of ankle fractures involving the posterior malleolus.

**Methods:**

A total of 51 patients with ankle fractures involving the posterior malleolus admitted to our hospital from January 2018 to December 2019 were selected. The patients were divided into 3D printing group (28 cases) and control group (23 cases). 3D printing was performed for ankle fractures, followed by printing of a solid model and simulation of the operation on the 3D model. The operation was then performed according to the preoperative plan, including open reduction and internal fixation via the posterolateral approach with the patient in the prone position. Routine x-ray and CT examinations of the ankle joint were performed, and ankle function was evaluated using the American Foot and Ankle Surgery Association (AOFAS) ankle-hindfoot score.

**Results:**

All patients underwent x-ray and CT examinations. All fractures healed clinically, without loss of reduction or failure of internal fixation. Good clinical effects were achieved in both groups of patients. The operation time, intraoperative blood loss and intraoperative fluoroscopy frequency in the 3D printing group were significantly less than those in the control group (*p *< 0.05). There was no significant difference between the two groups in the anatomical reduction rate of fractures or the incidence of surgical complications (*p *> 0.05).

**Conclusion:**

The 3D printing-assisted posterolateral approach is effective in the treatment of ankle fractures involving the posterior malleolus. The approach can be well planned before the operation, is simple to perform, yields good fracture reduction and fixation, and has good prospects for clinical application.

## 1. Introduction

Ankle fracture is the most common intra-articular fracture, accounting for 3.92% of all fractures (1). The stability and flexibility of the ankle joint is very important. Improper treatment may lead to traumatic arthritis, joint deformity or ankle dysfunction (2). Complex rotational trauma often leads to ankle fractures involving the posterior malleolus, with a high incidence, accounting for 7%–44% of all ankle fractures (3). Anatomically, the posterior malleolus is also called the posterior tubercle of the distal tibia, which plays a role in preventing the talus from moving backward (4). It cooperates with the lateral malleolus and the posterior tibiofibular ligament to maintain the stability of the ankle joint. Improper treatment of posterior malleolar fractures will lead to adverse consequences, such as a reduction in the contact area of the tibiotalar joint, an increase in joint stress, posterior displacement of the talus, and injury to the lower tibiofibular joint, which will destroy the stability of the ankle joint and eventually lead to traumatic arthritis, affecting the quality of life of patients (5).

There have been many clinical reports on the use of open reduction and internal fixation in the treatment of posterior malleolar fractures, with satisfactory clinical results. Surgical treatment requires precise anatomical reduction and firm, stable internal fixation. At present, there is no clear standard regarding the surgical indications for posterior malleolar fractures. It is generally believed that if a posterior malleolar fracture involves 25%–30% of the tibial articular surface and/or the fracture displacement exceeds 2 mm, the stability of the ankle joint will be affected, and open reduction and internal fixation of the fracture is required (6).

The concept of digital orthopedic precision has been used to plan and perform precisely individualized surgery, thus improving the accuracy and safety of surgery and reducing surgical trauma. 3D printing technology is a modern technology that focuses on digitalization and precision surgery and provides a bridge between real and virtual surgery. Computerized 3D models are the basis of 3D printing, which is used to construct real objects by layer deposition or bonding of powder or liquid materials. As a new technology, 3D printing has been rapidly promoted in orthopedics and other fields, especially in complex orthopedic surgeries involving the knee, shoulder, elbow and pelvis, and has yielded good clinical results (7, 8). 3D printing can also be used to assist in surgery for treating ankle fractures. Based on the preoperative x-ray and 3D CT images of patients with fractures, a virtual 3D model is constructed to quickly print an individualized fracture model; then, the specific fracture site, fracture severity, and degree of articular surface and soft tissue damage can be intuitively understood, allowing an individualized surgical plan to be formulated and the possible risks and prognosis of the surgery to be predicted. Research has confirmed that 3D printing-assisted surgical treatment allows not only exposure of the ankle fracture site but also preoperative determination of the fracture classification and selection of appropriate fixation methods and materials (9). Advanced preparation of these materials facilitates their direct use during surgery, and only fine adjustment is needed in cases of mismatching, which improves the accuracy of surgery and greatly reduces the operation time.

There is no consensus on the best surgical approach for treating ankle fractures involving the posterior malleolus. The existing surgical approaches include the posteromedial approach, posterolateral approach, and combined posterolateral-posteromedial approach, each with its own shortcomings. In this study, we reviewed 28 cases of ankle fractures involving the posterior malleolus treated from January 2018 to December 2019 with open reduction and internal fixation through the posterolateral approach with the assistance of 3D printing. Satisfaction with anatomical fracture reduction and the American Foot and Ankle Surgery Association (AOFAS) score on follow-up were determined through postoperative imaging examinations to evaluate the clinical efficacy of the 3D printing-assisted posterolateral approach in the treatment of ankle fractures involving the posterior malleolus.

## 2. Materials and methods

### 2.1. General information

The study protocol was approved by the Ethics Committees of Guizhou Provincial People's Hospital. Patients with ankle fractures involving the posterior malleolus treated at our hospital from January 2018 to December 2019 were selected. Random number table grouping method was used to divide into 3D printing group (28 cases) and control group (23 cases). 3D printing group: Before the operation, a 3D model of the fracture was printed and a simulated fracture reduction and fixation operation was carried out to guide the reduction and internal fixation of fractures during the operation, as well as the determination of appropriate bone plate and steel plate prebending. In the control group, surgery was performed in a traditional manner without 3D printing. All patients signed informed consent forms. In the 3D printing group, among the 28 patients with ankle fractures involving the posterior malleolus, 17 were males, and 11 were females; the patient age range was 45.86 ± 12.50 years. The causes of injury were as follows: fall from a height, 11 cases; traffic accident, 9 cases; sprain due to a fall, 7 cases; and trauma involving a heavy object, 1 case. According to the classification system developed by Lauge-Hansen for ankle fractures, there were 23 cases of supination and external rotation injuries and 5 cases of pronation and external rotation injuries. The control group consisted of 14 males and 9 females, with an average age of 49.65 ± 13.78 years. The causes of injury were as follows: fall from a height, 10 cases; traffic accident, 7 cases; and sprain due to a fall, 6 cases. There were 20 cases of supination and external rotation injuries and 3 cases of pronation and external rotation injuries.

All fractures were closed fractures. The patients underwent surgery 3–10 days after injury. The operations in both groups were performed by the same surgical team, including one operator and two surgical assistants. There was no significant difference between the two groups in terms of sex, age, injury factors, fracture type, or the time from injury to surgical treatment (*p* > 0.05).

The inclusion criteria were as follows: (1) history of trauma to the affected ankle joint; (2) specific signs of fractures, such as swelling, pain, deformity and abnormal movement of the affected ankle joint; (3) closed external rotation ankle fracture involving the posterior malleolus confirmed by x-ray and CT examination; and (4) fresh ankle fracture (within 2 weeks of injury).

The exclusion criteria were as follows: (1) pathological fracture; (2) open fracture; and (3) comorbidities, such as heart, lung, liver, kidney or other organ dysfunction or systemic malnutrition leading to an inability to tolerate surgery.

### 2.2. 3D data acquisition

3D image construction and 3D printing were performed using plain 64-row CT data (layer spacing, 0.5 mm; voltage, 120 kV; and current, 150 mA) imported into Mimics 3D reconstruction software. After editing images to construct the virtual model in Mimics, the virtual model was exported to the 3D printer in STL format, and the printing orientation was adjusted to print the solid model for surgical design.

### 2.3. Surgical methods

All patients were operated on in the prone position. After general anesthesia or epidural anesthesia was induced, the affected side was identified, an airbag tourniquet was placed, and routine sterilization and draping were performed. The midpoint of the lateral malleolus and the Achilles tendon was identified, and a longitudinal incision of approximately 7 cm–10 cm in length was created. The skin and subcutaneous tissues were incised layer by layer, and attention was given to protect the sural nerve and the small saphenous vein. Then, the fibularis longus and brevis tendons were retracted posteriorly, the end of the fibular fracture was directly reduced, and the lateral malleolus was fixed with a posterolateral plate. After the fibula was fixed, the fractured posterior malleolus was reduced. Fracture reduction and fixation were carried out according to the 3D-printed model prepared before the operation and the displacement of the fracture block during the operation. If the CT examination of the ankle joint before the operation showed no free fracture fragments in the articular cavity, the fractured posterior ankle bone was directly reset. The reference point used for reduction was the tip of the posterior malleolus. For the top edge of the fracture, cannulated screws with a diameter of 4.0 mm or 3.0 mm were used to fix the posterior ankle. A total of 1–2 screws were used according to the size of the fracture. For elderly patients with obvious osteoporosis, a posterior buttress plate could be used. Intraoperative C-arm x-ray fluoroscopy was performed to ensure anatomical reduction of the posterior malleolus and lateral malleolus without the penetration of any screws into the joint cavity. The medial malleolus was reached by a small incision and fixed with 1–2 cannulated screws. For patients who still showed lower tibiofibular separation after trimalleolar fixation was completed, lower tibiofibular screw fixation was performed. During surgery, use an aspirator to drain bleeding to a dedicated device and alculate the bleeding amount.

Antibiotics were administered prophylactically for 24 h after the operation, mannitol was used to reduce swelling, and toe flexion and extension exercises were performed on the day of the operation. Regular outpatient review was performed after the operation to evaluate the surgical incision sites, fracture healing, ankle joint functional recovery, and complications, including wound infection, skin necrosis, fracture displacement recurrence, internal fixation loosening, detachment, fracture malunion, and delayed union.

### 2.4. Rehabilitation protocol

Patients were encouraged to engage in activities within 2 weeks after the operation and to combine passive activities with active training within 3–6 weeks. Walking with partial weight-bearing assisted by a brace was performed 6–8 weeks after the operation, according to x-ray and CT observation of fracture healing, and complete weight-bearing activities were started 12 weeks after the operation.

### 2.5. Outcome measures

The operation time and intraoperative blood loss were recorded. Follow-up plain x-ray and CT examinations were performed after the operation to assess the anatomical reduction of the posterior ankle fracture, fracture and joint alignment, and internal fixation. At the same time, using the AOFAS ankle-hindfoot score (10), functional recovery of the ankle joint was evaluated according to the patient's ankle pain severity, walking distance and gait, degree of impact on daily life, ankle joint stability, and range of ankle joint activity. The total score is 100 points; the higher the score, the better the ankle and hindfoot function. Ankle function scores were determined 12 months after the operation and were ranked as follows: excellent, 90–100 points; good, 75–89 points; acceptable, 50–74 points; and poor, less than 50 points.

### 2.6. Statistical methods

SPSS 21.0 statistical software was used for statistical analysis of the research results. The *t* test was used to compare quantitative data with a normal distribution between groups, and the Fisher exact probability method or chi square test was used to compare qualitative data between groups. *p *< 0.05 was considered to indicate a statistically significant difference.

## 3. Results

### 3.1. Comparison of operation time, intraoperative blood loss and intraoperative fluoroscopy frequency between the two groups

The operation time in the 3D printing group was 85.29 ± 5.96 min, while that in the control group was 97.52 ± 5.82 min. The intraoperative blood loss in the 3D printing group was 109.18 ± 8.94 ml, while that in the control group was 140.0 ± 11.24 ml. Similarly, the intraoperative fluoroscopy frequency also differed between the 3D printing and control groups. According to Table 1, the operation time, blood loss and intraoperative fluoroscopy frequency in the 3D printing group were significantly lower than those in the control group (*p* < 0.05).

**Table 1:** Comparison of operation time, bleeding volume and intraoperative fluoroscopy frequency between the two groups.

Group	Operation time (min)	Bleeding volume (ml)	Intraoperative fluoroscopy frequency
3D printing (28 cases)	85.29 ± 5.96*	109.18 ± 8.94*	4.00 (3.00–5.00)*
Control (23 cases)	97.52 ± 5.82	140.0 ± 11.24	6.00 (5.00–8.00)

* *p* < 0.05 (compared with control, *p* < 0.05).

### 3.2. Evaluation criteria for postoperative anatomical reduction

The deformity and displacement of the fracture were completely corrected to achieve anatomical reduction, with good fracture and joint alignment. In this study, according to the x-ray and CT examinations after the operation, All patients have good anatomical reduction. All fractures healed clinically, without loss of reduction or failure of internal fixation.

### 3.3. Incision healing after operation

One patient in the control group developed a necrotic wound margin accompanied by a small amount of exudation, which healed with improved wound dressing. The incisions of other patients healed well, without skin necrosis, infection, sinus formation, instrumentation exposure or other complications.

### 3.4. AOFAS ankle-hindfoot score

In the 3D printing group, at the last follow-up (12 months after the operation), the AOFAS ankle-hindfoot score was excellent in 21 cases, good in 5 cases, and average in 2 cases, for a rate of excellent and good scores of 92.86%. In the control group, the AOFAS ankle-hindfoot score was excellent in 16 cases, good in 5 cases, and average in 2 cases, for a rate of excellent and good scores of 91.30%. There was no significant difference between the two groups (*p *< 0.05) (Table 2).

**Table 2:** Comparison of the AOFAS ankle-hindfoot score between the two groups.

Group	Excellent	Good	Average	Rate of excellent and good scores
3D printing (28 cases)	21	5	2	92.86%
Control (23 cases)	16	5	2	91.30%

**Figure 1:**
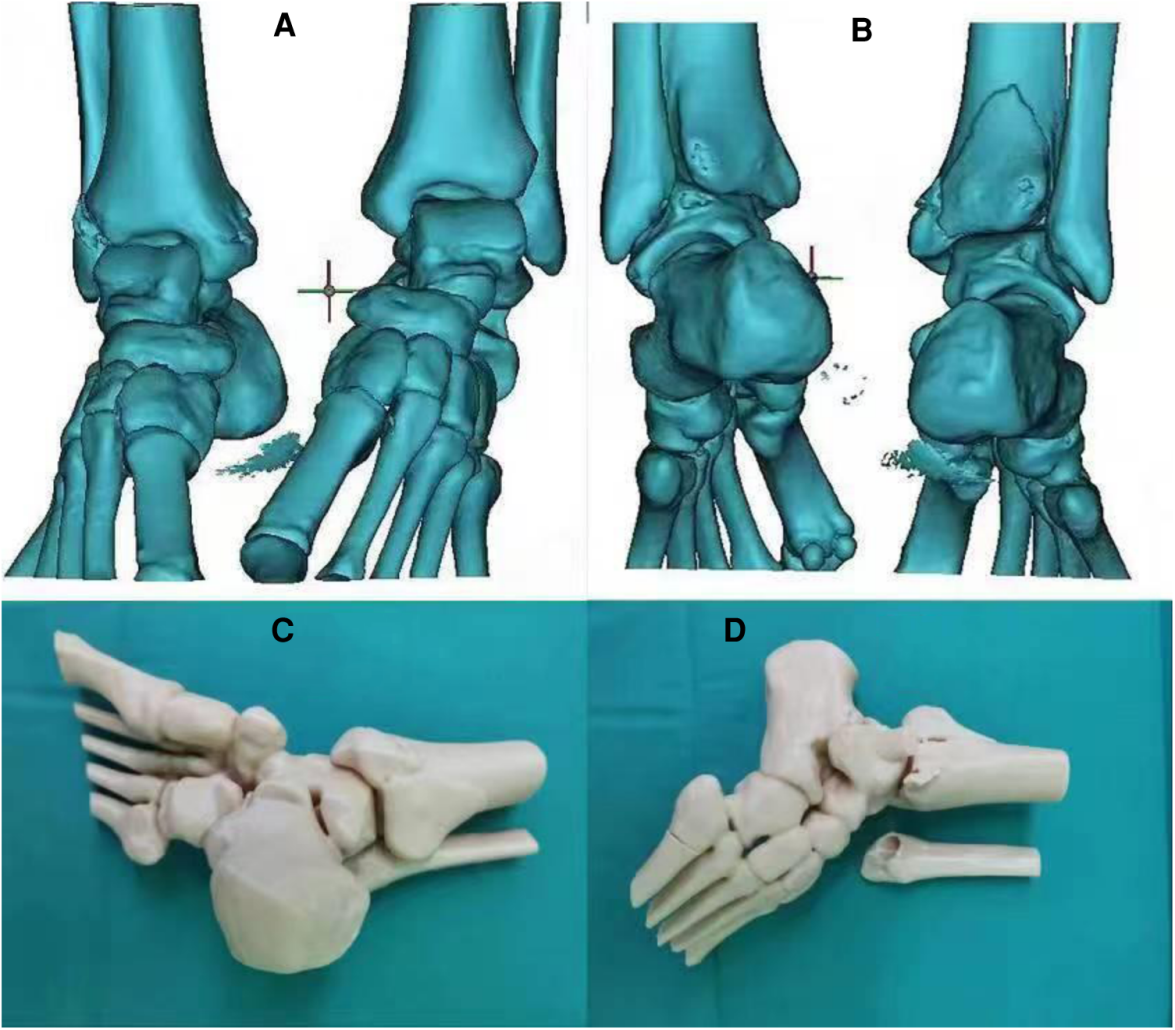
Preoperative 3D model of ankle fracture.

Figure 1 shows a 3D model before the operation, which clearly shows the outline of the ankle fracture and was very helpful for intraoperative fracture reduction and internal fixation.

**Figure 2:**
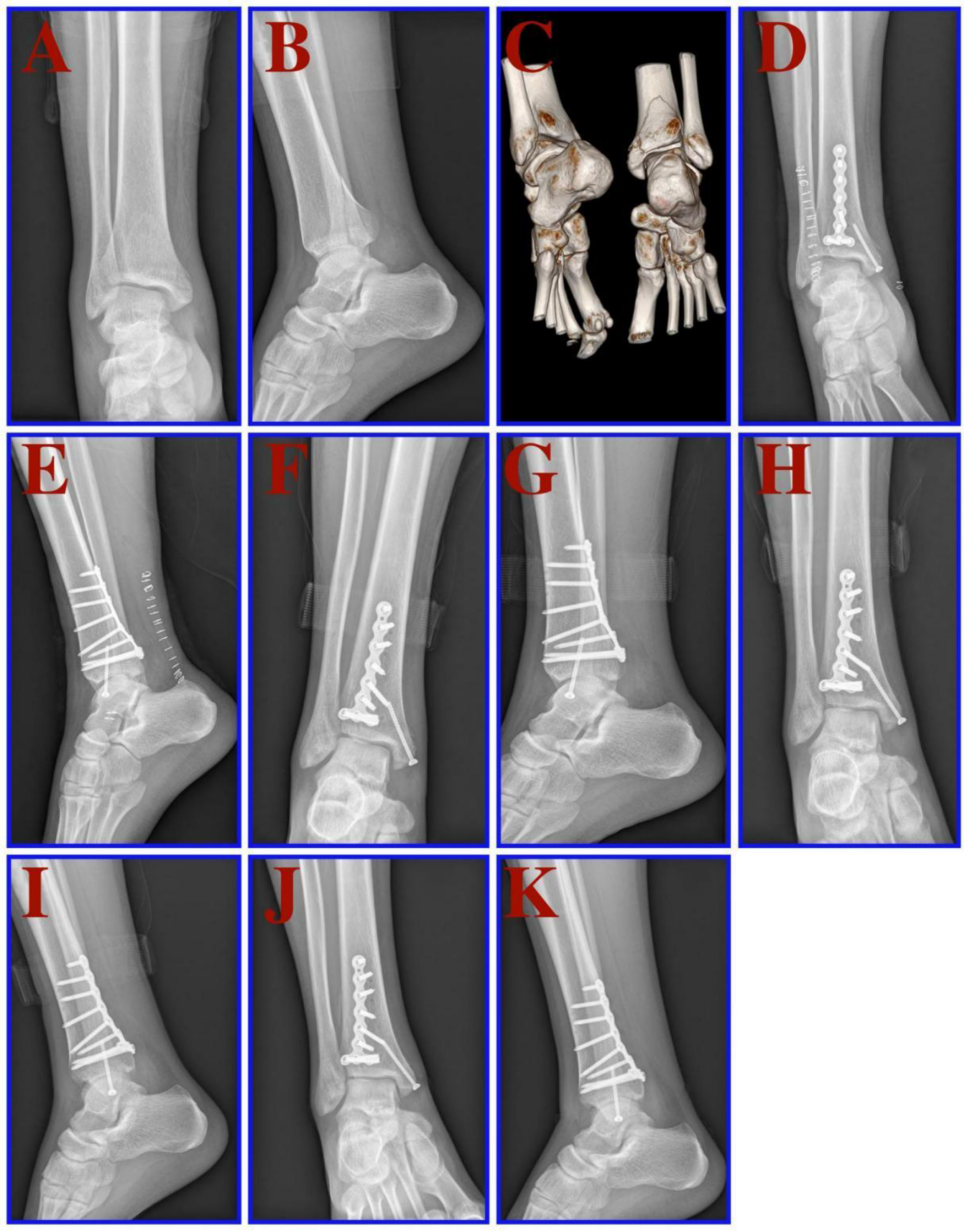
A 32-year-old male patient suffered from a bimalleolar fracture of the right ankle after a fall. (**A,B**) x-rays (front and side views) before the operation. **C**: Preoperative CT and 3D reconstruction. (**D,E**) x-rays (front and side views) 1 day after the operation. (**F,G**) x-rays (front and side views) one month after the operation. (**H,I**) x-rays (front and side views) three months after the operation. (**J,K**) x-rays (front and side views) six months after the operation.

Figure 2 (A–K) shows preoperative and postoperative images of a typical case of posterior malleolar fracture.

## 4. Discussion

The ankle joint bears most of the weight of the human body, and its stability depends on three ligament complexes, namely, the inferior tibiofibular complex, the medial complex and the lateral complex. Fractures are prone to occur when the stability of the ankle joint structure is disrupted (11). The posterior malleolus is the attachment point of the posterior tibiofibular ligament. Connection of the lateral malleolus through the ligaments is an important component of the anatomical and structural stability of the ankle joint; coordination between the lateral malleolus and the posterior tibiofibular ligament can reduce the stress of the tibiotalar joint and maintain the stability of the ankle. Fracture of the posterior malleolus is caused by forward impact of the posterior malleolus and backward impact of the talus under inertial action with reaction forces exceeding the bearing limit. When the posterior malleolus is fractured, the stability of the posterior talus and the integrity of the ankle joint are destroyed, the effective contact area of the tibiotalar joint is reduced, and the site of contact stress in the joint moves inward, resulting in accelerated cartilage wear on the articular surface, which in turn increases the possibility of traumatic ankle arthritis. If a posterior malleolar fracture is not effectively treated, it will affect daily walking abilities and reduce quality of life. Posterior malleolar fractures are intra-articular fractures. For such fractures, it is necessary to achieve accurate anatomical reduction and strong internal fixation as much as possible to restore the tightness of the ankle, continuity of reconstructed ligaments, and integrity of the articular surface, as well as to create conditions for early functional exercise after surgery, prevent posterior capsule contracture from limiting ankle joint dorsiflexion, and reduce the possibility of traumatic arthritis and ankle instability in the long term (12). Research has shown that the combination of posterior malleolar fractures with other malleolar fractures is an indicator of fracture complexity and trauma severity, which are closely related to the prognosis; additionally, the possibility of traumatic arthritis is high in such cases (13). Therefore, great attention should be given to ankle fractures involving the posterior malleolus.

At present, there is no clear standard regarding the surgical indications for posterior malleolar fracture block fixation. Some studies have found that if the posterior malleolar fracture block involves less than 10% of the articular surface and the lower tibiofibular joint ligament is complete, the stability of the ankle joint can be restored after fixation of the medial and lateral malleoli, with no significant difference between surgical and conservative treatment; thus, conservative treatment can be considered in such cases (14). In the 1960 s, some scholars conducted research on a large number of posterior malleolar fractures treated conservatively and found that when the posterior malleolar fracture involved more than 25% of the ankle joint surface, the anatomical structure of the ankle point was damaged, the posterior malleolus lost its ability to prevent posterior dislocation of the talus, and the ankle joint lost stability, leading to traumatic arthritis (15), which has become a commonly accepted surgical indication for posterior malleolar fractures and is recognized by most orthopedic doctors. Other scholars believe that when the posterior ankle fracture block involves more than 10% of the ankle joint surface, the ankle joint will lose stability, and the structure will be damaged, affecting the prognosis (16). Biomechanical research has also shown that when the posterior ankle fracture block affects more than 10% of the ankle joint surface, the original contact area of the tibiotalar joint decreases, increasing the incidence of traumatic arthritis (17). Therefore, for patients with posterior malleolar fractures, even if the fracture block is very small, great attention should be given to ensure the flatness of the distal tibial joint surface. Thus, we believe that the indications for open reduction and internal fixation of posterior malleolar fractures include the following: posterior malleolar fracture mass >25% of the ankle articular surface or fracture displacement >2 mm; posterior malleolar fracture mass >10% of ankle articular surface, vertical displacement ≥1 mm, or combination with lower tibiofibular syndesmosis injury or instability; and posterior ankle fracture block <10% of ankle joint surface (combination with lower tibiofibular syndesmosis injury or instability can be regarded as a relative surgical indication). All patients in this study with posterior malleolar fractures were treated with open reduction and internal fixation. Long-term follow-up revealed that good clinical effects were achieved in most of the patients. Therefore, for most posterior malleolar fractures, open reduction and internal fixation are necessary.

How to choose the surgical approach for the treatment of posterior malleolar fractures continues to be a difficult problem for orthopedic doctors. Posterior malleolar fractures are often accompanied by lateral or medial malleolar fractures. Selection of the appropriate surgical approach should consider not only the size and displacement of the posterior malleolar fracture block but also the treatment of medial and lateral malleolar fractures (18). A good surgical approach can simplify fracture reduction, facilitate reliable internal fixation, and create conditions for early postoperative functional joint exercise (19). In this study, we chose the posterolateral approach for the open reduction and internal fixation of fractures, mainly based on the following points: The posterior malleolar fracture block is generally located on the posterolateral side of the tibia near the lateral malleolus, and the posterolateral approach can expose the whole posterior malleolar fracture block and the posterior tibiofibular ligament, which not only facilitates soft tissue and hematoma removal from the fracture end but also improves the accuracy of anatomical reduction and is conducive to the postoperative recovery of patients. The posterolateral incision can fully expose both posterior and lateral malleolar fractures at the same time, and advanced consideration of the reduction and internal fixation of the lateral malleolar fracture block may allow sufficient exposure to be achieved while shortening the incision and reducing soft tissue damage. Finally, as the pollicis longus is innervated by the tibial nerve and the peroneus longus and peroneus brevis are innervated by the superficial peroneal nerve, surgery in the neuromuscular space will not cause postoperative muscle paralysis.

As traditional plain x-ray and CT examinations can be disrupted by bone block angles or overlap, it can be difficult to determine the direction of fracture displacement, especially for comminuted fractures around joints. Although 3D CT reconstruction can be used to observe fractures from multiple angles, it also provides a two-dimensional planar display (20), and the surgeon can only reset the bone according to a mental 3D model. This surgery is limited by the operator's clinical experience and operation skills, and the learning curve is long. In this study, we prepared a 3D-printed model of the ankle joint before surgery and used it to classify and fully simulate the reduction and fixation of ankle joint fractures. This method was conducive to reducing the difficulty and duration of surgery, intraoperative bleeding, the number of intraoperative fluoroscopy examinations, and the incidence of postoperative complications.

In this study, 3D printing technology was used to carry out preoperative design and surgical simulation for 28 patients with ankle fractures involving the posterior malleolus, and a detailed surgical plan was developed accordingly. During the operation, it was found that the physical model based on 3D printing technology could clearly display the position and size of the fracture block, be used to accurately simulate screw placement and accurately measure the length and angle of the screws and steel plate, and provide an intuitive and reliable reference for formulation of the surgical strategy. The results of fracture reduction and internal fixation were consistent with the preoperative simulation, and the virtual operation design was basically consistent with the real operation. More importantly, in this study, the operation time, bleeding volume and intraoperative fluoroscopy frequency in the 3D group were significantly less than those in the control group (*p* < 0.05), indicating that the application of digital technology combined with 3D printing-assisted reconstruction can significantly shorten the operation time and reduce the surgical trauma. Our research findings are consistent with reports in the literature reports. Yang et al. (21) evaluated the effect of 3D printing in the treatment of ankle fractures and its role in doctor-patient communication. The team randomly divided 30 patients with ankle fractures into a 3D printing group and a non-3D printing group. The results showed that the operation time and bleeding volume in the 3D printing group were significantly less than those in the non-3D printing group. More importantly, patient satisfaction was significantly higher in the 3D printing group than in the non-3D printing group. 3D printing is not only effective in helping doctors plan surgery but is also an effective tool for doctor-patient communication. Zhang et al. (22) also reached a similar conclusion regarding the 3D printing-assisted treatment of complex ankle joint fractures.

3D printing has several advantages. Through 3D printing technology, a 1:1 physical model can be created, which allows doctors to intuitively, stereoscopically and comprehensively understand the fracture, including the size of the fracture block and the degree of bone compression, and directly measure the fracture on the model. At the same time, patients can also gain a full understanding of their condition through observation of the fracture model, which is conducive to doctor–patient communication, improves patient compliance, and has a positive impact on patient satisfaction. During the operation, the attachment of internal fixation instrumentation to the bone surface is often less than ideal, or the screw angle may be poor, and many iterations of shaping or adjustment may be needed to achieve appropriate positioning (23). However, fracture reduction and titanium plate shaping can be carried out on the basis of a 3D model, which allows the titanium plate to be perfectly fixed to the fracture end after reduction, as the appropriate position, angle and length of the titanium plate and screw have already been determined. The accurate preoperative design and full understanding of the relationship between fracture blocks help to reduce excessive soft tissue stripping and protect the cutaneous blood supply during the operation, as well as simplify the operation process, and reduce the operation time, soft tissue exposure, bleeding and number of fluoroscopy examinations needed during the operation (24). The visual field is limited by the presence of blood vessels, nerves, muscles and soft tissues during surgery, especially surgery for reducing foot fractures, which is difficult. Before the operation, the fracture block and surrounding anatomy can be identified according to the model, which is helpful for the placement of instrumentation during the operation and anatomical fracture reduction (25). In addition, if bone grafting is needed during the operation, the extent of the bone defect can be accurately determined using the digital model before the operation.

While seeing the advantages of 3D technology, we should also be aware of its disadvantages and shortcomings. In this study, although compared with the control group, the 3D printing group shortened the operation time, reduced the amount of intraoperative bleeding and the number of intraoperative transmission, there was no statistical difference between the two groups in the anatomical reduction rate of surgery and the ankle function at the last follow-up (*p* > 0.05), and the 3D printing cost was high, which increased the economic burden of patients (26). In addition, due to the relatively complex 3D printing process, and the 3D printing solid model takes a long time and cannot effectively guide emergency surgery (27). Therefore, we may need to consider the advantages and disadvantages of 3D printing comprehensively, such as the complexity of fractures, the patient's economic affordability and the production cycle of 3D printing models in clinical application (28). Although there are many constraints on 3D printing technology, with the progress of 3D printing technology, the reduction of cost and the shortening of cycle, 3D printing will be more and more widely used in surgery.

However, there are some limitations to this study. The number of cases included in the study was small, the follow-up time was limited, and a power analysis was not performed. Therefore, further prospective research with a larger sample size and extended follow-up period is needed.

## 5. Conclusion

The preoperative planning and simulation operation function of 3D printing provides convenience for the treatment of ankle fractures involving the posterior malleolus, improves the accuracy during operation, shortens the operation time and reduces the trauma during operation. while surgery was done more quickly, with less fluoroscopic imaging, reduction quality did not differ appeared further research is needed to determine whether or not 3D printed modeling is cost effective, given that there was no difference in anatomic reduction or patient reported outcome scores.

## Data Availability

The original contributions presented in the study are included in the article, further inquiries can be directed to the corresponding author.
